# Bioactive Diterpenes from the Brazilian Native Plant (*Moquiniastrum pulchrum*) and Their Application in Weed Control

**DOI:** 10.3390/molecules26154632

**Published:** 2021-07-30

**Authors:** Fátima Vela, Simoni Anese, Rosa M. Varela, Ascensión Torres, José M. G. Molinillo, Francisco A. Macías

**Affiliations:** 1 Allelopathy Group, Department of Organic Chemistry, Institute of Biomolecules (INBIO), Faculty of Science, University of Cadiz, C/República Saharaui #7, 11510 Puerto Real, Spain; fatimavela22@gmail.com (F.V.); chema.gonzalez@uca.es (J.M.G.M.); famacias@uca.es (F.A.M.); 2 Federal Institute of Education, Science and Technology of Mato Grosso—Campus Campo Novo do Parecis, MT 235 Km 12, Campo Novo do Parecis 78360-000, Brazil; simoni.anese@cnp.ifmt.edu.br

**Keywords:** *Moquiniastrum pulchrum*, flavones, diterpenes, phytotoxic, coleoptiles, weeds

## Abstract

Even today, weeds continue to be a considerable problem for agriculture. The application of synthetic herbicides produces serious environmental consequences, and crops suffer loss of their activity due to the appearance of new resistant weed biotypes. Our aim is to develop new effective natural herbicides that improve the problem of resistance and do not harm the environment. This work is focused on a bioassay-guided isolation and the characterization of natural products present in *Moquiniastrum pulchrum* leaves with phytotoxic activity and its preliminary application in weeds. *Moquiniastrum pulchrum* was selected for two reasons: it is an abundant species in the Cerrado region (the second most important ecosystem in Brazil, after the Amazon)—the explanation behind its being a dominant species is a major focus of interest—and it has traditional employment in folk medicine. Six major compounds were isolated in this plant: one flavone and five diterpenes, two of which are described for the first time in the literature. Four of the six compounds exhibited phytotoxic activity in the bioassays performed. The results confirmed the phytotoxic potential of this plant, which had not been investigated until now.

## 1. Introduction

The area known as Cerrado boasts an ecosystem of immense biodiversity [1]. This vast region stretches through a total of 205 million hectares, and it is the second largest plant formation in Brazil [2]. It has undergone heavy deforestation and fragmentation as a result of the progressive agricultural expansion in this area [3,4]. The Cerrado contains more than 12,000 species of plants, many of which remain to be studied from a phytochemical point of view [5].

It is well known that allelochemicals are the secondary metabolites responsible for the interactions between plants [6]. These compounds can be released into the environment through four pathways: leaching, volatilization, exudation, or decomposition. They can be found in any of the plant parts, including its bark, leaves, or fruits among others [7]. We consider that understanding the main interactions between these secondary metabolites and their effect on weeds may be rather enlightening toward a better control of the weeds that affect the agricultural industry [8].

Until present, weed control has been mainly based on the use of synthetic herbicides [9]; however, although it is undeniable that this type of herbicides provides considerable benefits, we must also admit that the continuous application of synthetic herbicides comes along with a series of serious drawbacks, such as their lack of specificity, their heavy environmental impact, the undesired incorporation of herbicide residues to food products and the emergence of resistant biotypes [10]. Even if weed control may be an essential aspect for a crop to come through, the total suppression of weeds and the subsequent loss of biodiversity does not appear to be the right target to aim at. Furthermore, when a particular type of weed is fully eradicated from an area, frequently, a new and less controllable weed species takes over.

The complexity of this problem has encouraged research on alternative routes based on the synthesis of natural compounds. Over the last few years, natural products with phytotoxic activity have been studied as an alternative source of model compounds to be used for the production of a new generation of herbicides. Such natural products present a number of advantages when compared to synthetic herbicides given that they can be active at low concentration levels, they have a shorter life and, more importantly, their vast structural diversity would lead to a similar diversity regarding their modes of action [11].

There are many studies that provide new tools for their control in agricultural fields, whose phytotoxicity was evaluated in terms of wheat coleoptile elongation [12,13,14]. In this way, synergistic effects arising from allelochemical combinations were also measured with this bioassay, and these were found to enhance the efficacy of these compounds more than on their own, increasing the knowledge regarding the phytotoxic activity and agricultural potential of plant extracts [15].

*Asteraceae* is a family with 1535 genera and between 23,000 to 32,000 species, with a wide distribution. [16] In Brazil, we can find approximately 300 genera and 3000 species [17,18]. This family is rich in secondary metabolites used as storage compounds or chemical defense [5]. *Moquiniastrum* belonged to the genus *Gochnatia* until recently, when molecular studies enabled its elevation to the level of genus. The new genus *Moquiniastrum* (Asteraceae) includes 21 species of shrubs and occasionally trees, distributed mainly in Brazil in the main domains phytogeographics that include the Cerrado [19]. The species belonging to this genus are known for their importance in traditional medicine and have been reported for the treatment of respiratory diseases and anti-inflammatory, antispasmodic, and antimicrobial action [20].

Previous research studies have reported that trees from the genus *Moquiniastrum* have been often employed in folk medicine as a generic treatment for infections, inflammations, or pain. Until now, 14 species of *Moquiniastrum* have been investigated, and sesquiterpenic lactones, sesquiterpenes, diterpenes, triterpenes, flavonoids, and coumarins have been identified as their main compounds [21]. Some of those compounds have also been evaluated for pharmacological activity (antibacterial and anti-inflammatory) [20,22]; however, their phytotoxic activities are yet to be determined.

Although we do not fully understand the mechanisms used by plants to exert an influence on the growth of the other plants in their vicinity, such mechanisms are expected to play a considerable role regarding the development of an efficient weed control method [23]. Due to their need to adapt physically to their ever-changing environment, plants develop their own self-defense systems.

As a consequence, they become a very interesting source of a large number of structurally diverse organic compounds, many of which turn out to be allelopathic agents [24]. As already documented by many research papers on allelopathic interactions, such compounds may represent a source of molecules that could lead to the development of new organo-chemical products with the potential to be used as cost-effective and environmentally friendly weed-control agents [7,25,26].

Two facts support the study of this plant in this article: its great abundance in the Cerrado areas where it is endemic [19,27] and its use in folk medicine [20]. The abundance of this angiosperm could actually be explained by the allelochemical compounds that it produces and that make it a predominant species in its environment. The fact that it possesses pharmacological properties indicates that it has active principles that could have other types of activities, such as phytotoxic. Such phytotoxic activities still remain to be properly evaluated.

Therefore, this work intends to evaluate the phytotoxic potential of *M. pulchrum* leaf extracts and their compounds. Thus, by means of a biodirected study of leaf extracts allowed the isolation and structural determination of six major compounds described for the first time in this plant as follows: a flavone and five kaurane-type diterpenes differentiated by hydroxyl and acetyl group substitutions. Two out of these five diterpenes are described in the literature for the first time: moquinian A (**4**) and moquinian B (**5**). All the six compounds were tested by wheat etiolated coleoptile bioassay.

The flavonoid (**1**) and three of the diterpenes (**2**–**4**) exhibited significant growth inhibition levels, with **3** presenting the best activity profile. Based on the activities in the coleoptile bioassay, compounds **1**–**5** were selected for a more specific phytotoxicity bioassay intending to evaluate their activity on the germination and elongation of the roots and stems of specific weed seeds (*Echinochloa crus-galli* and *Amaranthus viridis*).

*A. viridis* proved to be more sensitive to the inhibitory effects of diterpene compounds **2**–**5**, with diterpene **4** showing an even higher phytotoxic activity than the commercial herbicide Logran^®^. In view of these promising results, the investigation on the allelopathic properties and the active principles of this plant could represent a significant contribution to a deeper knowledge on the native wild flora from the Cerrado region. Furthermore, these new compounds could represent the basis for the development of novel and more natural and environmentally friendly herbicides that could contribute to preserving the biodiversity.

## 2. Results and Discussion

### 2.1. Extract Separation Process

**Figure 1:**
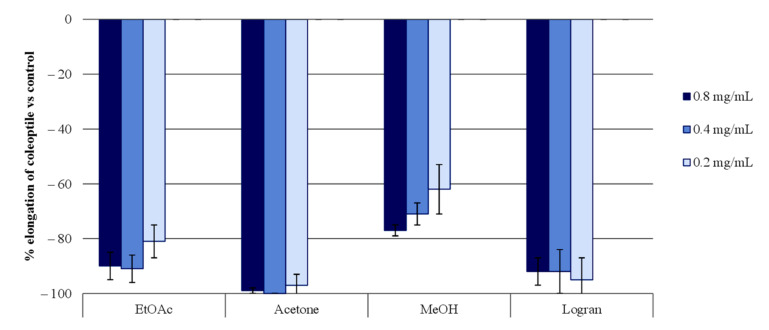
The bioactivities obtained in the etiolated wheat coleoptile bioassay for the *Moquiniastrum pulchrum* leaf extracts. Values are expressed as the percentage difference with respect to the control. Each bar is the mean ± standard deviation.

Dried leaves (1500 g) of *Moquiniastrum pulchrum*, defatted with hexane, were extracted with ethyl acetate, acetone, and methanol. Extracts at concentrations of 0.8, 0.4, and 0.2 mg/mL were subjected to a wheat ethiolate coleoptile bioassay (Figure 1). The wheat coleoptile bioassay has the advantages of being rapid and sensitive to detect phytotoxins [28]. This bioassay showed a higher inhibition with the acetone extract with a value of 99% at 0.8 mg/mL, and the activity was held upon dilution. The EtOAc fractions exceeded 80% inhibition at the three concentration levels tested (0.8, 0.4, and 0.2 mg/mL), while the three dilutions of MeOH at the same concentration levels reached just over 60% inhibition activity.

**Figure 2:**
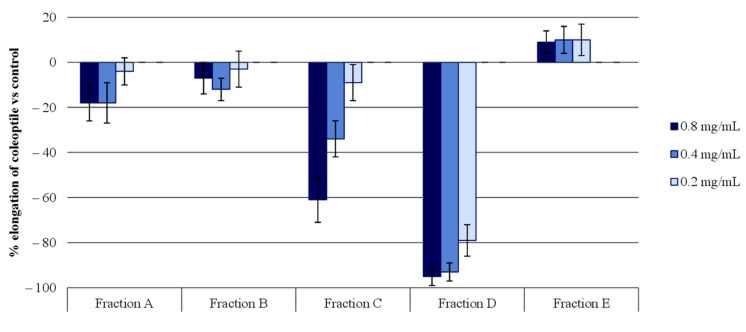
The bioactivities obtained in the etiolated wheat coleoptile bioassay for the fractions obtained from the acetone extract of *Moquiniastrum pulchrum* leaves. Values are expressed as the percentage difference with respect to the control. Each bar is the mean ± standard deviation.

After the acetone extract was obtained, the chlorophyll was removed by means of an RP-18 column containing a mixture of MeOH:H_2_O in increasing polarity. Five fractions were obtained (A–E) and evaluated by means of the wheat etiolated coleoptiles bioassays (Figure 2). The activity corresponding to this extract is represented by fraction D. This fraction exhibited the highest percentage of wheat coleoptile elongation inhibition, with values as high as 95% and 92% for the 0.8 and 0.4 mg/mL concentrations, respectively, and 80% for the 0.2 mg/mL one.

**Figure 3:**
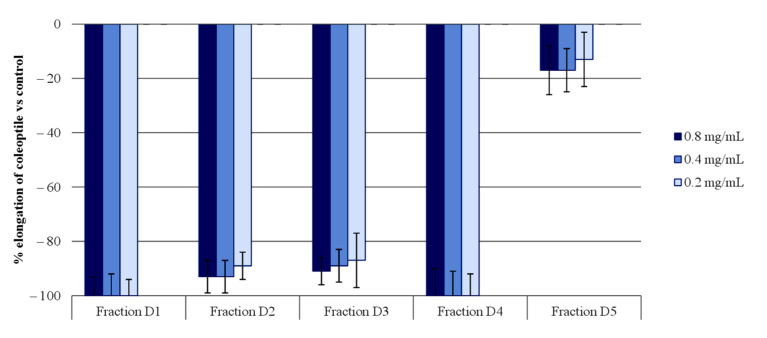
The bioactivities obtained in the etiolated wheat coleoptile bioassay for the subfractions obtained from fraction D of *M. pulchrum* leaves. Values are expressed as the percentage difference with respect to the control. Each bar is the mean ± standard deviation.

Fraction D was then subjected to normal phase column chromatography (silica gel) using a mixture of the solvents DCM:EtOAc in increasing polarity. Five fractions of interest were obtained (D1–D5) and evaluated in coleoptile bioassays (Figure 3). According to the results, all the fractions, with the exception of fraction D5, exhibited high inhibition levels. Fractions D1 and D4 showed better inhibition activity profiles than the initial fraction (D), as the activity remained steady after dilution. These two fractions reached 100% inhibition at the three concentration levels tested (0.8, 0.4, and 0.2 mg/mL). On the other hand, the fractions D2 and D3 also reached around 90% inhibition levels at the same three concentration levels.

### 2.2. Isolation of Phytotoxic Compounds

These initial results confirm that the fractions D1–D4 of the *M. pulchrum* leaf extracts displayed activity in the wheat coleoptile bioassay. The isolation of the components from these fractions and the evaluation of their bioactivity could provide a better understanding of the ability of *M. pulchrum* as an abundant and dominant species in the Cerrado ecosystem. This should facilitate the discovery of new mechanisms for crop protection. In order to move further in this study, the fractions D1 and D2 were chromatographed on a silica gel column using chloroform:acetone in increasing polarity. Similarly, the fractions D3 and D4 were chromatographed on a silica gel column but using DCM: EtOAc. This process resulted in subfractions (D1) D1.1 to D1.4; (D2) D2.1 to D2.3; (D3) D3.1 to D3.3; and (D4) D4.1 to D4.4. These subfractions were purified, and the products were characterized.

**Figure 4:**
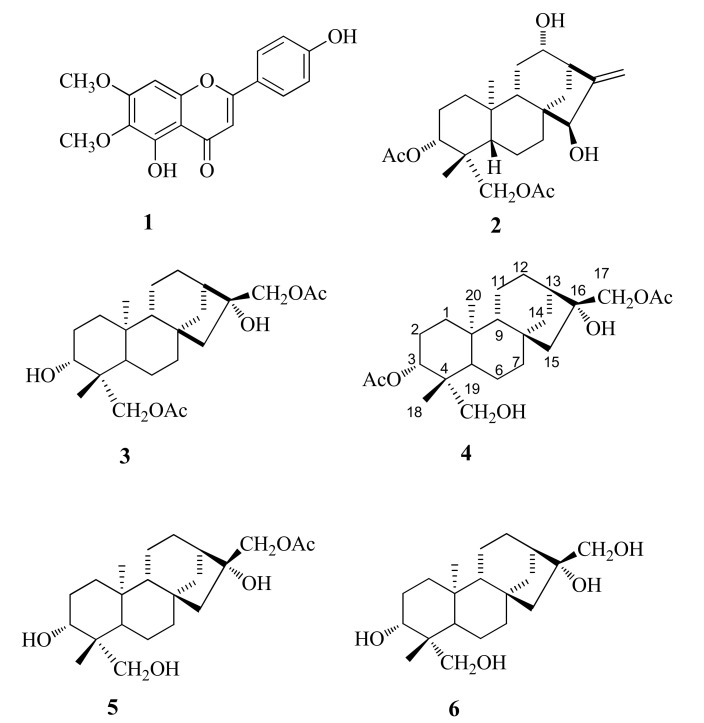
Structures of flavonoid (**1**) and diterpenes (**2**–**6**) isolated from fraction D of *M. pulchrum* leaves.

The subsequent chromatographic separations of the subfractions led to the isolation of the following compounds: one flavonoid (**1**) and five diterpenes (**2**–**5**) (Figure 4). The chemical profile of the extracts disclosed the presence of a high proportion of diterpenes. Several biological activities associated to diterpenes have already been described, including anti-inflammatory, antifungal, and antibacterial activities [29,30]. These compounds had not been previously isolated from *Moquiniastrum pulchrum*, and compounds **4** and **5** were described for the first time in the literature.

The flavonoid (**1**) and diterpenes (**2**, **3,** and **6**) isolated were identified by comparing their spectral properties (IR, MS, ^1^H NMR, and ^13^C NMR) against the published data. In this way, compound 1, the major product from active fraction D1 (but also present in the other active fractions: D2, D3, and D4), was isolated as a yellow solid, and was identified as cyrsimaritin [31] (Figure 4).

The isolation study of fraction D produced three known diterpenes (compounds **2** and **3** from D2 and **6** from D6) of the kaurane type as crystalline solids that corresponded to: *ent*-3α,19-diacetoxykaur-12α,15β-dihydroxykaur-16-ene (**2**) [32], *ent*-17,19-diacetoxy-*3*α,16α-dihydroxykaurane (**3**) [32], and *ent*-3α,16α,17,19-tetrahydroxykaurane (**6**) [33].

Compound **4** (52.25 mg) was isolated from subfraction D2.2 as a crystalline solid. Its mass spectrum showed a molecular ion at *m*/*z* 445.2568 [M + Na]^+^, which is consistent with the molecular formula C_24_H_38_O_6_Na. This compound is very similar to compound **3** (on the left side in Figure 4), with the only difference being the substitution of an acetyl group by a hydroxyl group between C-3 and C-19.

The ^13^C-NMR spectrum of compound **4** (Table 1) exhibited the presence of 24 skeletal carbons, of which, four were Me, ten were CH_2_, four were CH, and six were quaternary carbons. Two low-field signals at δ 171.2 and 169.8 ppm represented two carbonyl carbons of two ester groups. Then, one doublet at δ 82.8, one singlet at δ 79.8, and two triplets at δ 68.4 and 63.8 supported the presence of four oxygen-bonded carbons in the molecule.

The 500 MHz ^1^H-NMR spectrum (Table 1) displayed singlets of two acetates and two methyls. Of the two acetoxy groups, one was secondary equatorial and attached to C-3 as shown by a dd (*J* = 11 and 6 Hz at δ 4.59), while the second was primary (*sa*, δ 4.20) on C-17. The chemical shifts in the ^13^C NMR spectrum indicated these functionalizations at C-3 and C-17 [33], which was confirmed by HSQC spectroscopy. An OH was located at a methylene position attached to C-19 as shown by a d (*J* = 12 Hz at δ 4.12) and a t (*J* = 11 Hz at δ 3.36), while the sixth oxygen atom of the molecule had to be located as a hidroxyl on C-16 to explain the fact that none of the methylene protons under the acetate was further coupled.

Therefore, the compound would correspond to a kaurane-type diterpene with two hydroxyl groups and two acetyl groups. The acetyl groups were located on C-17 (δ 68.4) and C-3 (δ 82.8) and the hydroxyl groups on C-16 (δ 79.8) and C-19 (δ 63.8). This was deduced from the correlation between the signal δ 4.59 (H-3) with both the carbonyl signal from acetyl group (δ 169.8) and with the methyl group on C-18 (δ 22.4) that could be observed from the HMBC spectrum. Finally, the missing singlet signal from methyl 19, which is typical of the kaurane skeleton, and the presence of two signals in the oxygen-bound proton region at δ 4.12 and δ 3.36 appeared to indicate that the hydroxyl group was on C-19. The HSQC spectrum confirmed this assumption.

In addition to the multiplicity data, the stereochemistry of the C-3 and C-4 centers were determined through Nuclear Overhauser Effect (NOE) experiments. The irradiation of the H-3 signal gave a NOE effect (Nuclear Overhauser Effect) with the methyl signal at H-18β, which indicates that the acetyl group on C-3 is in the α position. H-19 gave a NOE effect with the H-20 α-signal, which indicates that the hydroxyl group on C-19 is at the α position.

Finally, the NOE effects observed between the signals from H-15α with H-14 and from H-15β with H-17, determined that, on C-16, the hydroxyl group is at the α position, and the methylene 17 with acetyl is at the β position. Consequently, all the data for this compound indicate that the structure is formulated as 3α,17-diacetoxy-16α,19-dihydroxykaurane, as shown in Figure 4 (Information on compound **4** is showed in the Supplementary Material). Thus, compound **4** is described for the first time in the literature and is given the name *moquinian A*.

Compound **5** (162.84 mg) was isolated as a crystalline solid from subfraction D 4.4. Its mass spectrum showed a molecular ion *m*/*z* at 403.2466 [M + Na]^+^, which is consistent with the formula C_22_H_36_O_5_ Na. In the ^13^C-NMR spectrum, 22 signals were observed (Table 1) of which, three were Me, ten were CH_2_, four were CH, and five were quaternary carbons. One low-field signal at δ 171.0 ppm represented the carbonyl carbon of an ester group. In addition, in the ^1^H-RMN spectrum, the signal found at δ 2.06 ppm confirmed that it is an acetyl group.

The ^1^H-RMN spectrum showed a close similarity with the spectrum of compound **4** (Table 1), which indicates that a kaurane type diterpene is involved, but now it presents one acetyl group and three hydroxyl groups. The H-3 proton shift appears at δ 3.30 ppm, which is typical of a geminal proton to a hydroxyl group, while, in the previous compound, it was at δ 4.59, being characteristic of geminal proton to an acetyl group.

Similarly, in the ^13^C-NMR spectrum, C-3 at δ 79.7 ppm indicates the presence of a carbon with hydroxyl group, whereas the typical shift at C-3 of the acetyl group appeared at δ 82.8 (compound **4**). Furthermore, in the HMBC spectrum, the signal at δ 3.30 (H-3) correlated with the signal at the C-18 methyl carbon (δ 22.0). The NOE effects (Nuclear Overhauser Effect) were the same as those of the previous compound; therefore, we can say that the substituents have the same relative orientation. Thus, the structure of compound **5** was determined as 17-acetoxy-3α,16α,19-trihydroxykaurane, as can be seen in Figure 4 (Information on compound **5** is showed in the Supplementary Material). Compound **5** is described for the first time in the literature and is given the name *moquinian B*.

### 2.3. Wheat Coleoptile Bioassays on Isolated Compounds

**Figure 5:**
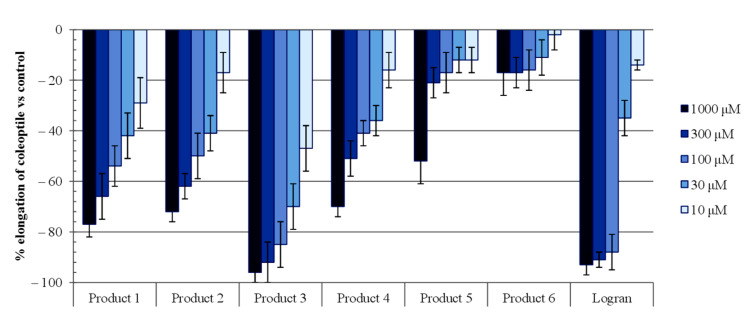
The effects of compounds (**1**–**6**) from *Moquiniastrum pulchrum* and triasulfuron sherbicide (Logran) on the elongation of etiolated wheat coleoptiles. Values are expressed as the percentage difference from the control. Each bar is the mean ± standard deviation.

The bioactivities of the isolated products (**1**–**6**) were evaluated by means of wheat coleoptile bioassays within the concentration range from 10 to 1000 µM. The results are shown in Figure 5.

Compound **1**, corresponding to flavone, showed an inhibitory activity of 77% for the most concentrated dilution (1000 μM), and a good activity profile when diluted. Among the activities of flavonoids, it has been demonstrated that they can be employed as antioxidants, enzyme inhibitors, pigments capable of absorbing harmful wavelengths (mainly in the UV region), protective agents against fungal invasions, and plant growth regulators, among other uses [34].

Numerous diterpenes are located at the leaf cuticle, and they have previously been described as part of the plants’ defensive system against insects and infectious agents [35]. As for the kaurane-type diterpenes, i.e., products **2**, **3**, **4**, **5**, and **6**, their activity varied depending on the type of substituent and the position where it is located. Product **3** showed the highest activity, with an inhibition percentage higher than 85% for the three highest concentrations tested (1000, 300, and 100 μM), and a good activity profile with dilution even better than that of the commercial herbicide Logran^®^. This was followed by product **2**, which reached an inhibitory activity of 72%, and compound 4 with 70%, at their highest concentration levels (1000 μM).

Similarly to what happened with compound **3**, the activities of these two compounds decreased uniformly with dilution. The remaining diterpenes (**5** and **6**) were the least active. The location of the two acetyl as well as the two hydroxyl groups that could be found in the three most active diterpenes (**2**–**4**) could explain this higher inhibitory capacity, which reached the maximum levels when the acetyl groups were on C-17 and C-19. These data were confirmed by the IC_50_ (Table 2), where product **3** displayed better values than even the commercial herbicide Logran^®^.

### 2.4. Phytotoxic Bioassay on the Most Active Compounds

**Figure 6:**
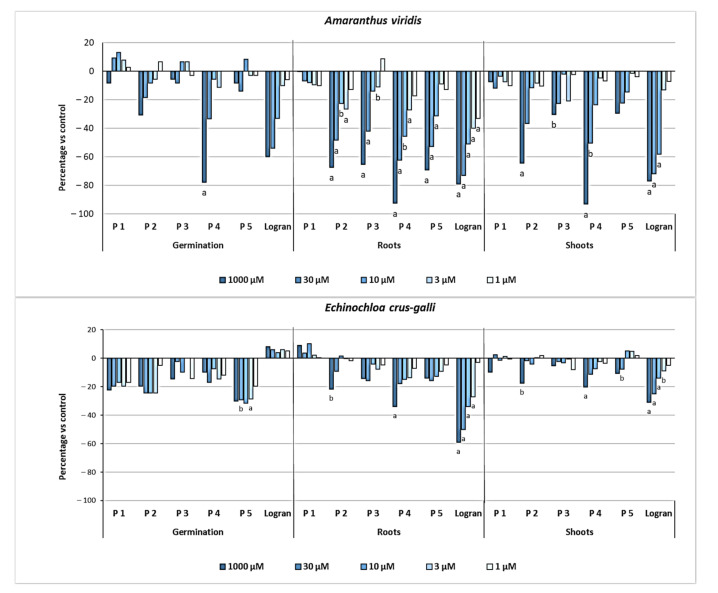
The effects of the compounds (**1**–**5**) obtainned from *M. pulchrum* leaves and of triasulfuron herbicide (Logran) on the germination and growth of weeds. Values are expressed as the percentage difference with respect to the control. Significance levels *p* < 0.01 (a) or 0.01 < *p* < 0.05 (b).

According to the inhibition values in Figure 5, compounds **1**–**5** presented a high activity on coleoptiles. For this reason, they were selected for a more specific phytotoxicity bioassay where their activity against the germination and elongation of roots and stems of weed seeds could be evaluated. For such phytotoxicity bioassays, two weeds were selected, namely *Echinochloa crus-galli* and *Amaranthus viridis* (Figure 6), which are known to be quite harmful for certain important crops, such as rice and corn, with serious weed control difficulties [36].

*A. viridis* was more sensitive to the inhibitory effects of the diterpene compounds **2**–**5**, in contrast with their poor activity against the weed *E. crus-galli*. See Figure 6. Flavonoid compound **1** (cyrsimaritin) did not display any activity against either of the two weeds tested. As mentioned above, some of the activities described regarding flavonoids are antioxidant, while others are mainly focused on the protection of the plants [34]. Nevertheless, methoxylated flavonoids have not shown any phytotoxic activities.

Compound 4 showed a higher inhibitory activity than the other compounds against the germination of *A. viridis* seeds, with an inhibition close to 80%, which is even better than that of the commercial herbicide tested at the 1000 µM concentration. *A. viridis* root growth was the parameter that registered the most evident effect from diterpenes **2**–**5**.

More specifically, compound 4 was again the one to show the strongest inhibitory activity; at over 90% when tested at the highest concentration level (1000 µM) and with a very consistent activity profile with the dilutions reaching 62%, 46%, and 27% when the dilutions were 30, 10, and 3 µM, respectively. This compound was followed by the diterpenes **2**, **3,** and **5**, all of which showed consistent activity profiles against root growth, with inhibitions close to 70% at the 1000 μM concentration.

As for shoot length, *A. viridis* was sensitive only to the inhibitory effects of the diterpenes **2** and **4**. Again, diterpene **4** showed the best inhibitory activity with 93% at the highest concentration tested, while compound **2** only reached 65% inhibition at the same dilution.

To summarize, in the phytotoxic bioassay, the diterpene moquinian A (compound **4**) was the one to contribute the most to the phytotoxic activity exhibited by the plant with respect to all the parameters evaluated on *A. viridis*. Diterpenes **2**, **3,** and **5**, which had been isolated in greater amounts from the leaves, showed good levels of inhibitory effects, particularly against the growth of the weed roots. In diterpenes **2**, **3**, and **4** the two acetyl groups and the two hydroxyl groups present in their structures once again correlated with a difference in activity.

## 3. Material and Methods

### 3.1. General Experimental Procedures

The infrared spectra (IR) were recorded on a Spectrum two spectrophotometer, serial number 98,863 by Fourier transform. (FT-IR) (Perkin-Elmer, Waltham, MA, USA). The nuclear magnetic resonance spectra (RMN) were acquired on a 500 MHz spectrometer. (Agilent, Palo Alto, CA, USA). The chemical shifts are given in ppm with respect to the ^1^H residual signals: the CHCl_3_-d_1_ (δ 7.25), MeOH-d_1_ (δ 3.30) and DMSO-d_1_ (δ 2.49), and the ^13^C signals refer to the solvent signal (δ 77.0), (δ 49.0), and (δ 39.5), respectively.

The mass spectrometry (MS) was carried out by means of a Synapt spectrometer G2 UPLC-QTOF ESI (Waters, Milford, MA, USA). The High Performance Liquid Chromatography (HPLC) was performed on an HPLC chromatograph (Merck-Hitachi, Tokyo, Japan) with refractive index detection. Silica gel 0.060–0.200 60 A from Acros Organics (Geel, Belgium) and Lichroprep RP18 (40–63 µm) from Merck (Darmstadt, Germany) were used for column chromatography.

The Thin Layer Chromatography (TLC) was performed on TLC Silica gel 60 F_254_ aluminum foils and TLC Silica gel 60 RP-18 F_254S_ aluminum foils from Merck (Darmstadt, Germany). The column used for the HPLCs was a semipreparative 250 × 10 mm column with identification LiChroCART 250-5 RP-18 (10 µm) Merck (Darmstadt, Germany). The ultrasound extractions were performed using an ultrasonic bath (360 W, J. P. Selecta, Barcelona, Spain) in three 15 min series.

### 3.2. Organic Solvents

Hexane (Hx), methanol (MeOH), dichloromethane (DCM), ethyl acetate (EtOAc), acetonitrile (AcN), and acetone (Hipersolv Chromanorm for HPLC) were purchased from VWR International (Radnor, PA, USA). MagniSolv chloroform-d_1_ (min. 99.8% deuteration degree), methanol-d_1_ (min. 99.8% deuteration degree), and DMSO-d_1_ (min. 99.8% deuteration degree) for the NMR spectroscopies were supplied by Merck (Darmstadt, Germany).

### 3.3. Preparation of Extracts and Isolation of Their Compounds

The *Moquiniastrum pulchrum* leaves were collected in July 2017 from the Cerrado biome reserve owned by the Federal University of São Carlos, São Paulo state—Brazil (22°02′ S and 47°52′ W). The species was identified by comparison with a specimen deposited in the Herbarium collection (SPSC) at the Federal University of São Carlos. A voucher specimen was deposited at this herbarium with the registration code 8708. After collection, the leaves were dried in ovens for 72 h at a temperature of 40 °C and ground by means of an industrial mill.

The starting point was 1500 g of dried and ground *Mosquiniastrum pulchrum* leaves. Of this, 250 g was defatted by means of hexane and extracted using an ultrasound-assisted procedure. Three extractions of 15 min each were made. The dry material was divided into smaller powder portions (of 250 g), which were subjected to the extraction processes by the same method using the following solvents: EtOAc acetone and MeOH. The resulting extracts were used for the wheat etiolated coleoptiles bioassays.

Considering that the acetone extract was the most active, the remaining leaves (1250 g), which were previously defatted, were divided into 15 g portions of plant material, and each portion was extracted using acetone in the same ultrasonic bath as above (3 × 250 mL). This process yielded 285.3 g of material. The chlorophyll was removed from the acetone extract using a reversed-phase silica gel column with MeOH/H_2_O mixtures in increasing MeOH concentration order; namely 20%, 40%, 60%, 80%, and 100%.

Finally, DCM was passed through the column to remove all the chlorophyll. Five fractions of chlorophyll free extracts were obtained with decreasing order of polarity: A (1.2 g), B (2.4 g), C (7.2 g), D (13.5 g), E (4.2 g), and DCM (3.5 g). These fractions were subjected to wheat etiolated coleoptile bioassays, where fraction D proved to be the most active (Figure 2).

Fraction D (13.5 g) was separated by column chromatography using a DCM/EtOAc mixture of increasing polarity from 0 to 100% with each time increasing 10% AcOEt. The following five subfractions were obtained: D1 (1.3 g), D2 (2.9 g), D3 (0.9 g), D4 (1.7 g), and D5 (1.0 g). These subfractions were again subjected to wheat coleoptile bioassays where all of them, with the exception of fraction D5, were confirmed to be active fractions (Figure 3). The four active fractions D1–D4 were separated chromatographically.

Fractions D1 (1.3432 g) and D2 (2.8514 g) were separated by column chromatography using a chloroform/acetone solvent mixture at increasing polarity, giving rise to several subfractions: (D1) D1.1 to D1.4 and (D2) D2.1 to D2.3. Subfractions D1.4 and D2.1 gave rise to compound **1** (96.6 mg). Subfraction D2.2 (0.7363 g) was purified by HPLC (reverse phase semi-preparative column) using a MeOH/AcN/H_2_O solvent mixture (27.5:27.5:45 *v*/*v* flow rate: 3 mL/min) to yield three subfractions: D2.2.1–D2.2.3. Subfraction D2.2.1 was identified as compound **2** (218.51 mg), D2.2.2 as compound **3** (135.4 mg), and D2.2.3 as compound **4** (52.25 mg).

The fractions D3 (0.86676 g) and D4 (1.7497 g) were separated by column chromatography using a DCM/EtOAc solvent mixture at increasing polarity and produced several subfractions as follows: (D3) D3.1 to D3.3 and (D4) D4.1 to D4.4. Subfraction D3.1 again gave rise to compound **1** (19.6 mg). Subfraction D3.3 (0.1871 g) was purified by HPLC (reverse phase semi-preparative column) using a MeOH/AcN/H_2_O solvent mixture (32:32:36 *v*/*v* flow rate: 3 mL/min) and produced compound **5** (100.14 mg). Subfraction D4.2 (0.1884 g) again yielded compound **5** (62.7 mg) as the majority.

Subfraction D4.4 (0.095 g) was separated by column chromatography using a mixture of the solvents DCM/AcOEt at increasing polarity and produced four subfractions: D4.4.1-D4.4.4. Subfraction D4.4.4 was purified by HPLC (reverse phase semi-preparative column) using a mixture of the solvent MeOH/AcN/H_2_O (23.5:23.5:5.3 *v*/*v* flow rate: 3 mL/min) and yielded compound **6** (24.7 mg). The structures of the isolated compounds (Figure 4) were determined by comparison with literature data from different spectroscopic experiments for all of them. All the isolated compounds were evaluated by coleoptile bioassays, and the most active ones were also subjected to phytotoxicity bioassays.

### 3.4. Spectroscopic Data of the New Compounds

3α,17-Diacetoxy-16α,19-dihydroxykaurane, moquinian A, (**4**). White crystalline solid; [α]^20^_D_ = −41.5° (c 1.0, MeOH); IR ν_max_ (KBr) cm^−1^; 3460 (OH); 1710, 1740 (C=O). ^1^H NMR (CDCl_3_, 500 MHz) data, see Table 1; ^13^C NMR (CDCl_3_, 125 MHz) data, see Table 1; positive-ion HR-TOF-ESIMS *m*/*z* 445.2568 [M + Na]^+^, (calcd for [M + Na]^+^, 445.2566).

17-Acetoxy-3α,16α,19-trihydroxykaurane, moquinian B, (**5**). White crystalline solid; [α]^20^_D_ = −32.8° (c 1.0, MeOH); IR ν_max_ (KBr) cm^−1^; 3370 (OH); 1700 (C=O). ^1^H NMR (MeOD, 500 MHz) data, see Table 2; ^13^C NMR (MeOD, 125 MHz) data, see Table 1; positive-ion HR-TOF-ESIMS *m*/*z* 403.2466 [M + Na]^+^ (calcd for [M + Na]^+^, 403.2460).

### 3.5. Coleoptiles Bioassay

The extracts, fractions, subfractions, and products were subjected to wheat etiolated coleoptile bioassays. This type of test is commonly used to evaluate the sensitivity of wheat to a wide range of bioactive substances [37].

*Triticum aestivum* L. seeds were pregerminated on Whatman paper No. 1 in 140 mm diameter Petri dishes for 72 h away from the light and at 22 ± 1 °C. Approximately 100 seeds were submerged into 15 mL of deionized water. After 72 h, the coleoptile fragments of 4 mm in length closest to the first two millimeters from the coleoptile tip were cut with a guillotine, selecting the tissue with the greatest elongation capacity. The coleoptiles were handled under green light to avoid photosynthesis.

The samples for the bioassays were dissolved in DMSO (0.1%) and diluted in phosphate-citrate buffer containing 2% sucrose at pH 5.6. Three control samples were used for reference as follows: buffer with DMSO, buffer alone, and Logran^®^ herbicide (59% terbutryn and 0.6% triasulfuron). The concentrations were 0.8, 0.4, and 0.2 mg/mL for the extracts and fractions, and 1000, 300, 100, 30, and 10 µM for the compounds. Each dilution was tested in triplicate.

Each bioassay was carried out using five coleoptiles and 2 mL of extract, buffer, or Logran^®^. The tubes were rotated at 6 rpm using a Stuart Scientific SC2-type culture rotor for 24 h at 25 °C in the dark. After 24 h, the coleoptiles were placed on a dark paper sheet photographed and measured using the application Photomed^®^. The results from the bioassays were expressed as percentages of the negative control sample.

### 3.6. Phytotoxic Bioassay

The most active compounds were assessed for their phytotoxic activity on two weed species that represent serious problems for agriculture: barnyard grass (*Echinochloa crus-galli*, *Poaceae*) and slender amaranth (*Amaranthus viridis, Amaranthaceae*). These bioassays were carried out on Whatman No. 1 paper in 50 mm diameter Petri dishes. The seeds were germinated and grown in aqueous solutions at a controlled pH (pH = 5.6), employing a 10 mM solution of 2-[*N*-morphino]ethanolsulfonic acid (MES), adjusted by means of a 1 M solution of NaOH.

The product solutions were prepared at the following concentrations: 1000, 300, 100, 30, and 10 µM using DMSO as co-solvent at a final concentration of 0.5% (*v*/*v*). These solutions were added to the Petri dishes containing the seeds under study (20 seeds per plate), and each test consisted of four replicates (a total population of 80 seeds) and 1 mL of solution, per plate. Each plate was sealed with Parafilm. The seeds were incubated at 25 °C for 7 days in the dark inside a growth chamber. After that period, the plates were stored at −10 °C for 24 h, to stop the growth of the seedlings and for later measurement.

The herbicide used as internal reference, Logran^®^, is applied at 0.4–0.6 kg/ha (equivalent concentration of 10,000 µM) doses as a pre- or post-emergence herbicide in barley and wheat crops against annual broadleaf weeds and as a pre-emergence herbicide against certain grass types.

The activity of the product was expressed as the percentage variation in root and stem growth and seed germination, with respect to that displayed by the control sample, where only the MES (2-[*N*-morphino]ethanolsulfonic acid) and 0.5% DMSO (*v*/*v*) buffer solution was used. The germination rate, root, and shoot length were recorded by means of the application Fitomed^®^ [38]. The results are presented as the percentage difference with respect to the control sample, where zero represents the control data, the positive values represent stimulation, and the negative values represent inhibition.

### 3.7. Statistical Analysis

The data from the bioassays were statistically analyzed using Welch’s test, where 0.01 and 0.05 were established as the significance levels. The half maximal inhibitory concentration (IC_50_) values were calculated from non-linear regression with the GraphPad Prism 5 package (San Diego, CA, USA).

## 4. Conclusions

Six compounds were isolated for the first time from the most active fraction of *M. pulchrum* leaves: one flavone (**1**) and five kaurane-type diterpenes (**2**–**5**), with two of them being described for the first time in the literature: moquinian A (**4**) and moquinian B (**5**). All of them were evaluated for bioactivity on etiolated wheat coleoptiles, and we confirmed their inhibitory capacity, which, for three of them, was comparable to that of the commercial herbicide Logran^®^. Subsequently, compounds **1**–**5** were tested in a phytotoxicity bioassay on weed seeds (*Echinochloa crus-galli* and *Amaranthus viridis*).

*A. viridis* was more sensitive to the inhibitory effects of diterpenes **2**–**5**, with diterpene **4** showing a higher phytotoxic activity than that of the commercial herbicide Logran^®^. The abundance of this angiosperm in Cerrado savannah areas, where it is endemic, could be explained by its production of allelochemical compounds that allow it to be predominant over other species in the same environment. Such active principles were confirmed to present phytotoxic activity against one of the most harmful weeds in agriculture: *A. viridis*.

This study on the allelopathic properties of the active principles of *M. pulchrum* represents a significant contribution to the scientific understanding of the interactions between the native wild flora in the Cerrado region and, more specifically, cast light on the competitive advantage attained by certain species. In addition, certain compounds, such as compound **4**, which is described for the first time in the literature and named moquinian A, was proven to be an ideal candidate for further studies aiming at developing novel and more environmentally friendly herbicides.

## Figures and Tables

**Table 1:** ^1^H NMR and ^13^C NMR spectroscopic data for compounds **4** and **5**.

	4 ^a,c^			5 ^b,c^	
Position	*δ*_H_ [*J* in Hz]	*δ*_C_	Position	*δ*_H_ [*J* in Hz]	*δ*_C_
1a	1.87 (1H, *dd*, *J* = 13; 3, 5 Hz)	36.8	1a	1.90 (1H, *dd*, *J* = 13; 3, 5 Hz)	36.5
1b	1.63 (1H, *ddd*, *J* = 13; 13; 3, 5 Hz)		1b	1.61 (1H, *ddd*, *J* = 13; 13; 4, 5 Hz)	
2	1.77 (2H, *m*)	23.5	2a	1.81 (1H, *m*)	27.0
3	4.59 (1H, *dd*, *J* = 11; 6 Hz)	82.8	2b	1.69 (1H, *m*)	
4	-	42.5	3	3.30 (1H, *dd*, *J* = 11; 5 Hz)	79.7
5	0.98 (1H, *da* *J* = 13 Hz)	55.8	4	-	42.2
6a	1.68 (1H, *m*)	20.5	5	0.89 (1H, *da*, *J* = 13 Hz)	55.4
6b	1.35 (1H, *m*)		6a	1.69 (1H, *m*)	20.2
7a	1.65 (1H, *m*)	41.8	6b	1.40 (1H, *m*)	
7b	1.47 (1H, *m*)		7a	1.64 (1H, *m*)	42.0
8	-	44.5	7b	1.48 (1H, *m*)	
9	0.99 (1H, *m*)	56.2	8	-	44.3
10	-	38.7	9	1.03 (1H, *m*)	56.6
11	1.53 (1H, *m*)	18.4	10	-	38.6
12	1.52 (2H, *m*)	26.0	11	1.58 (2H, *m*)	18.0
13	2.04 (1H, *m*)	45.9	12	1.56 (2H, *m)*	25.8
14a	1.83 (1H, *d*, *J* = 13 Hz)	38.2	13	2.08 (1H, *ta*, *J* = 3, 5 Hz)	45.4 38.2
14b	0.96 (1H, *dd*, *J* = 13; 5 Hz)		14a	1.85 (1H, *d*, *J* = 13 Hz)	38.2
15a	1.57 (1H, *d*, *J* = 15 Hz)	52.7	14b	0.89 (1H, *dd*, *J* = 13; 5 Hz)	
15b	1.48 (1H, *d*, *J* = 15 Hz)		15a	1.62 (1H, *d*, *J* = 15 Hz)	52.4
6	-	79.8	15b	1.44 (1H, *d*, *J* = 15 Hz)	
17	4.20 (2H, *sa*)	68.4	16	-	79.3
18	1.04 (3H, s)	22.4	17a	4.25 (1H, *sa*)	68.0
19a	4.12 (1H, *d*, *J* = 12 Hz)	63.8	17b	4.16 (1H, *sa*)	
19b	3.36 (1H, *t*, *J* = 11 Hz)		18	1.19 (3H, *s*)	22.0
20	0.98 (3H, *s*)	18.1	19a	4.07 (1H, *d*, *J* = 11, 5 Hz)	63.7
Ac	2.09 (3H, *s*) 2.07 (3H, *s*)	21.4 20.9	19b	3.36 (1H, *d*, *J* = 11, 5 Hz)	
Ac	2.07 (3H, *s*)	20.9	20	1.05 (3H, *s*)	17.3
C=O	-	171.2 169.8	Ac	2.06 (3H, *s*)	19.4
C=O	-	169.8	C=O	-	171.0

^a^ Data were measured at 500 MHz (^1^H NMR) and 125 MHz (^13^C NMR), (CDCl_3_). ^b^ Data were measured at 500 MHz (^1^H NMR) and 125 MHz (^13^C NMR), (MeOH). ^c^ Assignments were confirmed by ^1^H-^1^H-COSY, 1D-NOESY, HSQC, and HMBC experiments.

**Table 2:** The IC_50_ and clog*P* values calculated from the compounds (**1**–**6**) and Logran^®^ after the wheat coleoptile bioassay using a sigmoidal dose-response variable slope model.

Compound	IC_50_ [µg mL^−1^]	r^2^	clog*P*
**1**	56.79	0.925	2.247
**2**	76.29	0.927	2.801
**3**	11.33	0.998	3.155
**4**	276	0.919	3.773
**5**	1187	0.985	2.865
**6**	-	-	1.969
Logran^®^	39.45	0.950	-

## Data Availability

Not applicable.

## Supplementary Materials

The following are available online, compounds **4** and **5** NMR and HRMS spectra.

## Sample Availability

Samples of the compounds are available from the authors.
