# Supplementary material for: Bioactive Diterpenes from the Brazilian Native Plant (Moquiniastrum pulchrum) and Their Application in Weed Control
Source: Molecules. 2021 Jul 30;26(15):4632. doi: 10.3390/molecules26154632 (PMC8348793; doi:10.3390/molecules26154632)
Supplement: Supplementary file 1 [file molecules-26-04632-s001.zip › molecules-1307872-supplementary.pdf]

Supporting Information

Bioactive diterpenes from Brazilian native plant (*Moquiniastrum pulchrum*) and their application in weed control.

Fatima Vela[†], Simoni Anese[‡], Rosa M. Varela[†]*, Ascensión Torres[†]*, Jose M. G. Molinillo[†], Francisco A. Macias[†]

[†]Allelopathy Group, Department of Organic Chemistry, Institute of Biomolecules (INBIO), Faculty of Science, University of Cadiz, C/ República Saharaui #7, 11510 Puerto Real, (Cádiz), Spain

[‡] Federal Institute of Education, Science and Technology of Mato Grosso - Campus Campo Novo do Parecis, MT 235 Km 12, Campo Novo do Parecis, MT 78360-000, Brazil

* Author to whom correspondence should be addressed; E-mail: chon.torres@uca.es; rosa.varela@uca.es.
Phone: +34 956 01 27 90; Fax: +34 956 01 62 88.

S3 ^{1}H NMR spectrum of moquinian A (4) (500 MHz, Cl_3CD)

S3 ^{13}C NMR spectrum of moquinian A (4) (125 MHz, Cl_3CD)

S5 ^{1}H-^{1}H COSY spectrum of moquinian A (4) (125 MHz, Cl_3CD)

S5 HSQC spectrum of moquinian A (4) (125 MHz, Cl_3CD)

S7 HMBC spectrum of moquinian A (4) (125 MHz, Cl_3CD)

S7 TOF MS spectra of moquinian A (4)

S9 ^{1}H NMR spectrum of moquinian B (5) (500 MHz, MeOH)

S9 ^{13}C NMR spectrum of moquinian B (5) (125 MHz, MeOH)

S10 ^{1}H-^{1}H COSY spectrum of moquinian B (5) (125 MHz, MeOH)

S10 HSQC spectrum of moquinian B (5) (125 MHz, MeOH)

S11 HMBC spectrum of moquinian B (5) (125 MHz, MeOH)

S11 TOF MS spectra of moquinian B (5)

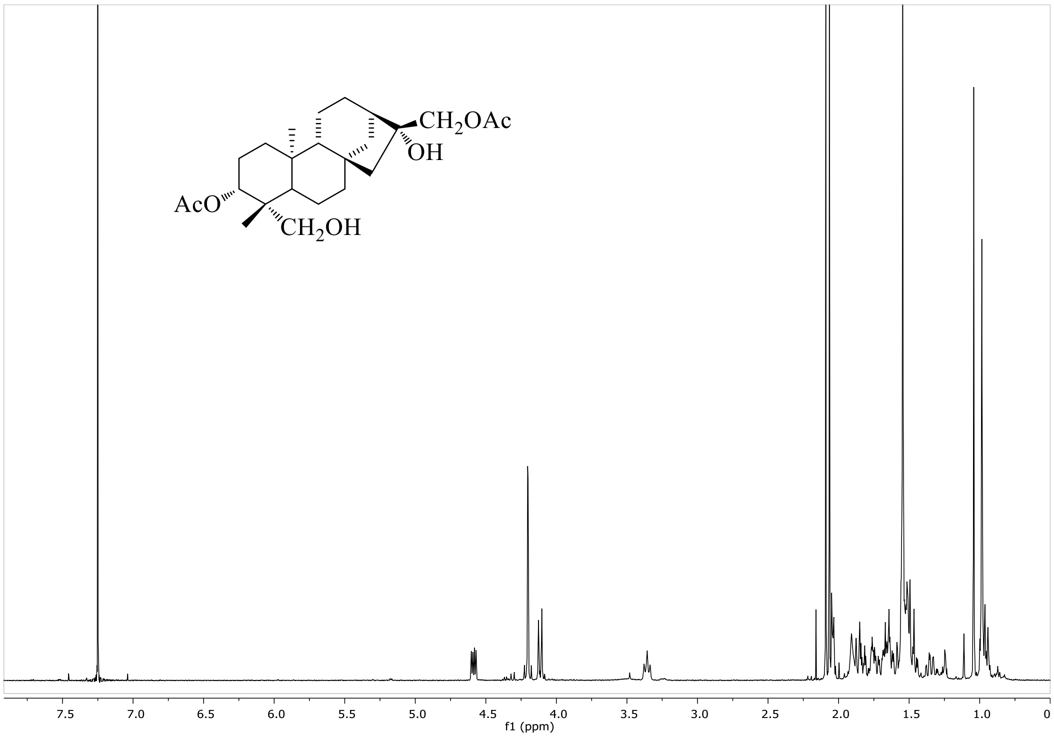

^{1}H NMR spectrum of moquinian A (4) (500 MHz, Cl_3CD)

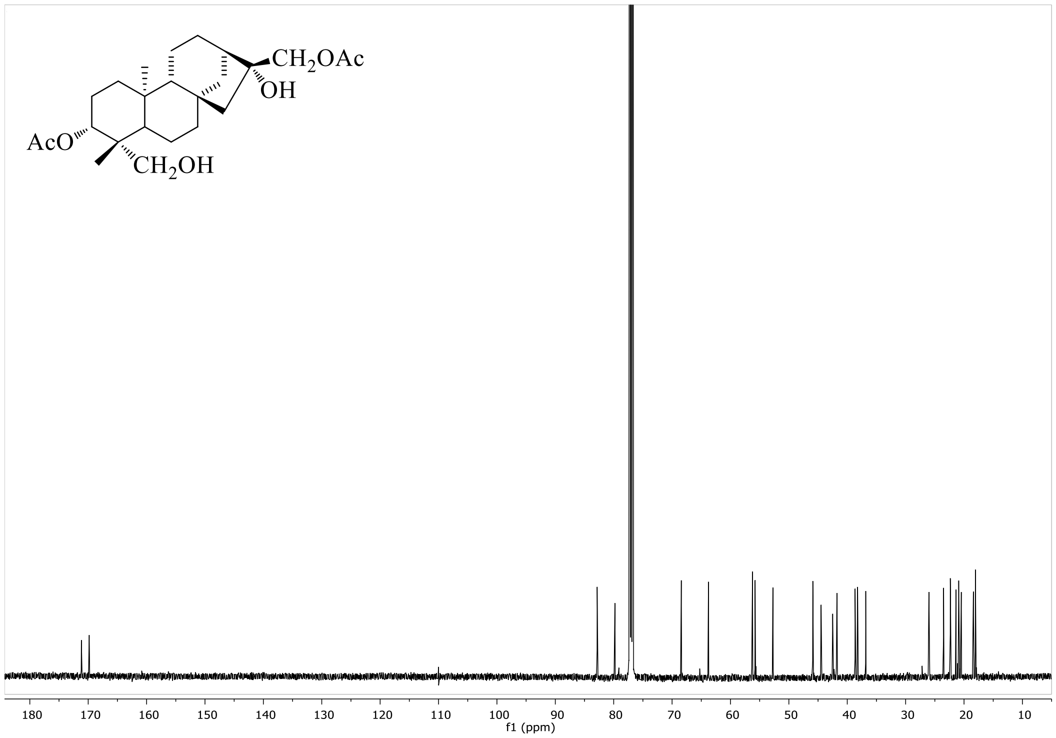

^{13}C NMR spectrum of moquinian A (4) (125 MHz, Cl_3CD)

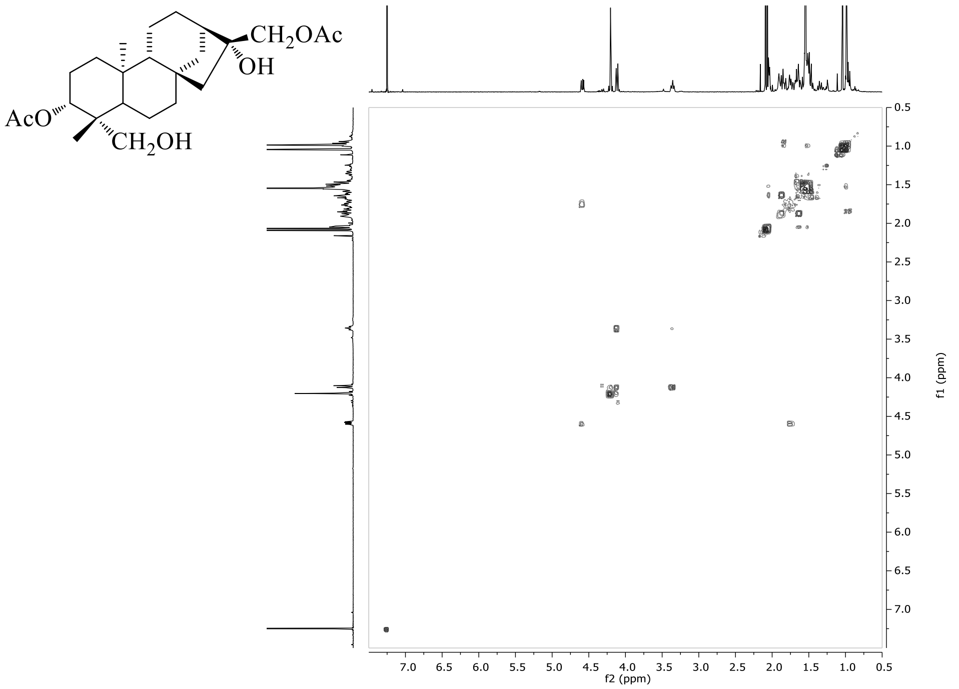

^1H-^1H COSY spectrum of moquinian A (4) (125 MHz, Cl_3CD)

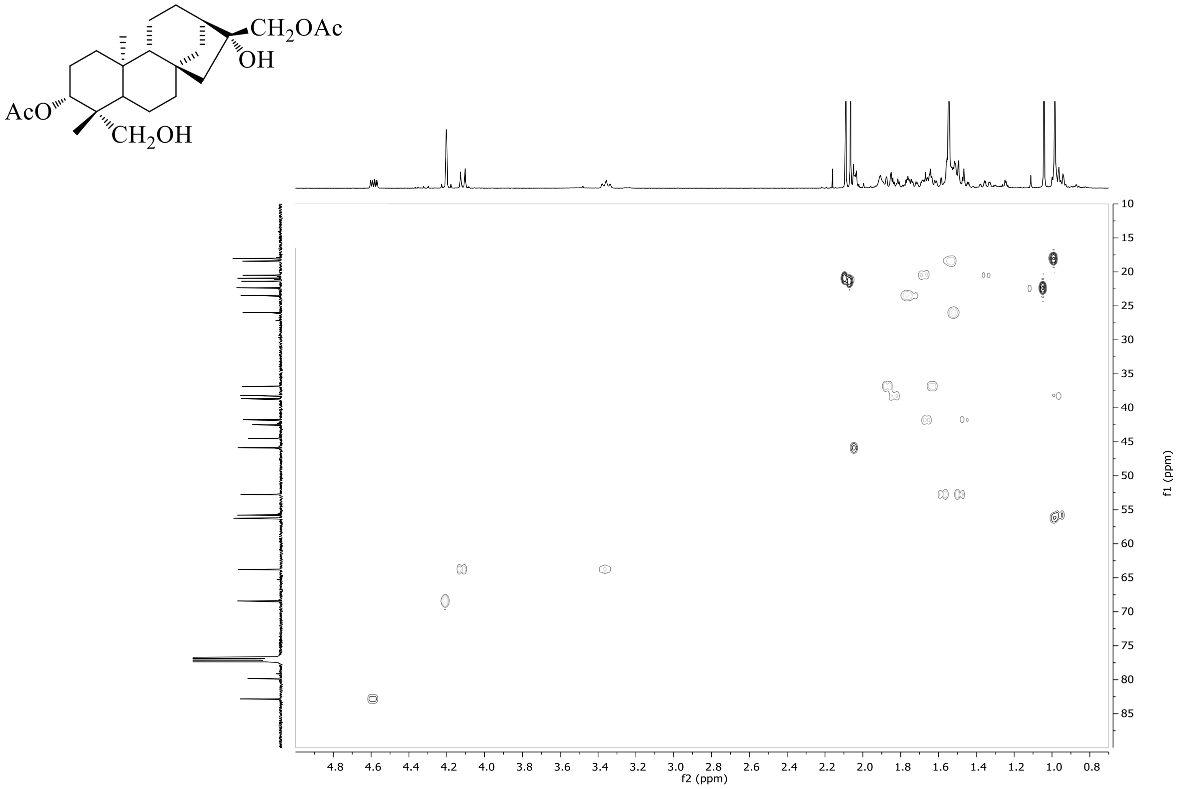

HSQC spectrum of moquinian A (4) (125 MHz, Cl_3CD)

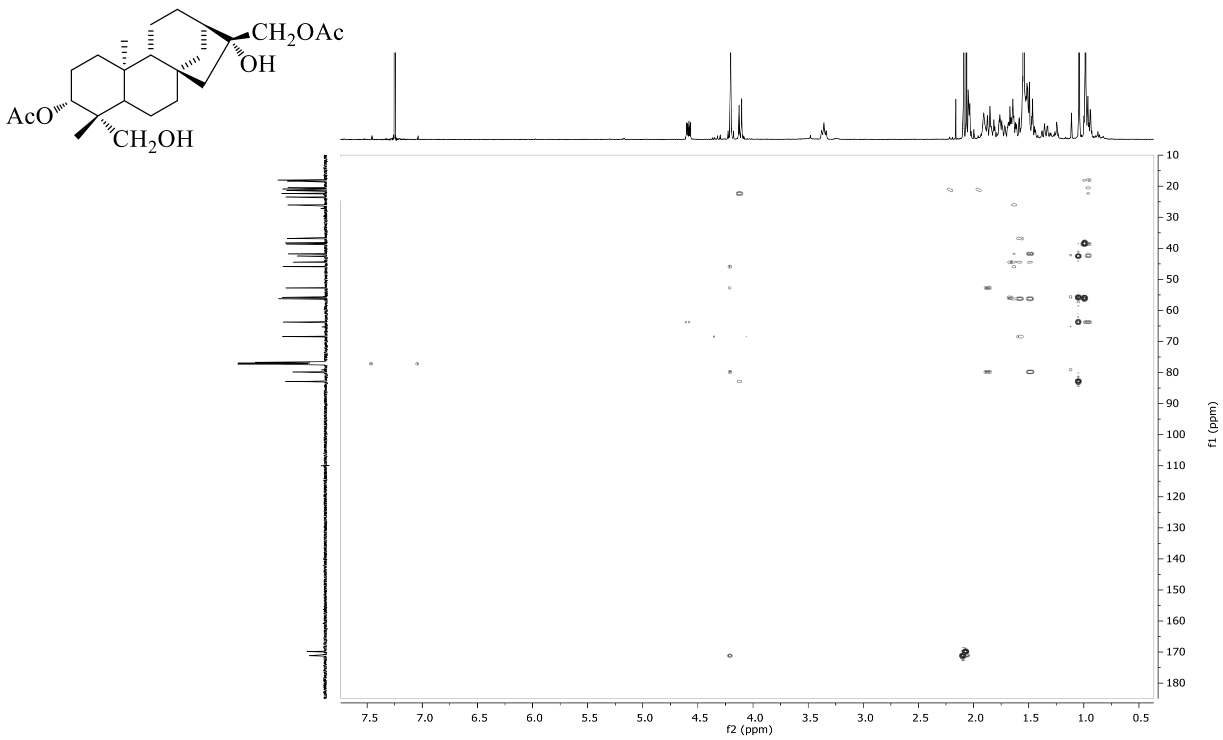

HMBC spectrum of moquinian A (4) (125 MHz, Cl3CD)

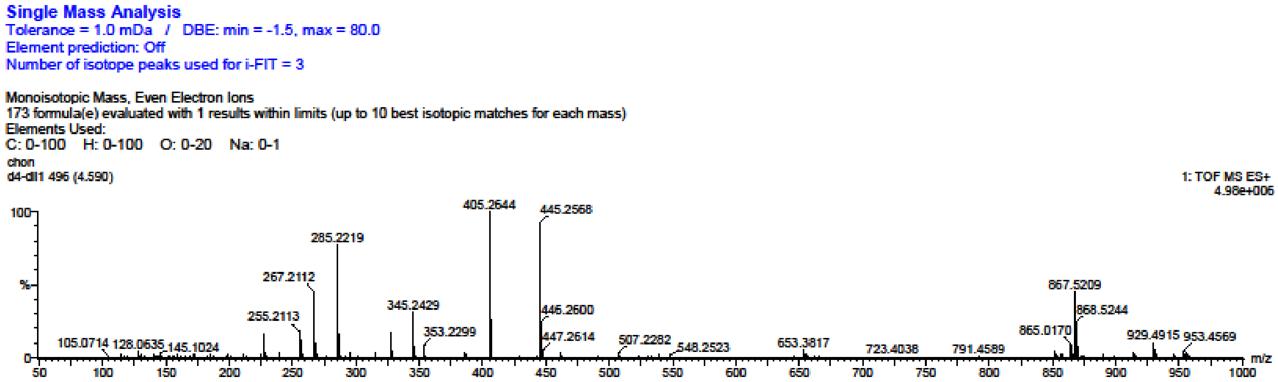

Minimum:				-1.5				
Maximum:		1.0	10.0	80.0				
Mass	Calc. Mass	mDa	PPM	DBE	i-FIT	Norm	Conf(%)	Formula
445.2568	445.2566	0.2	0.4	5.5	1233.5	n/a	n/a	C24 H38 O6 Na

TOF MS spectrum of moquinian A (4)

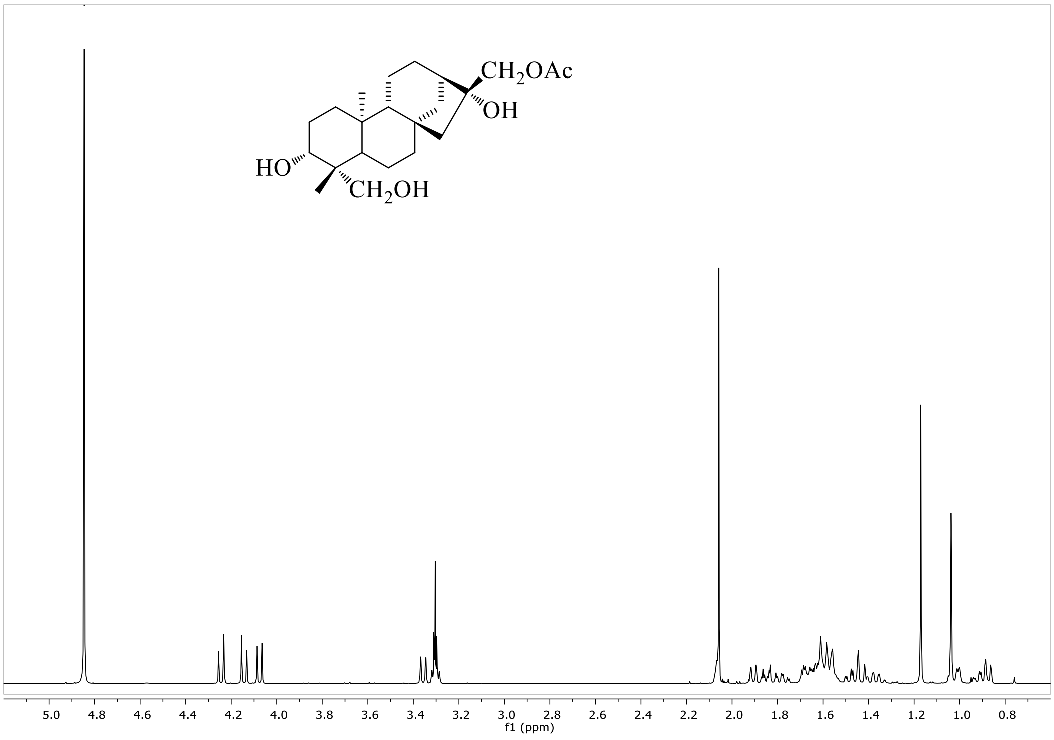

^{1}H NMR spectrum of moquinian B (5) (500 MHz, MeOH)

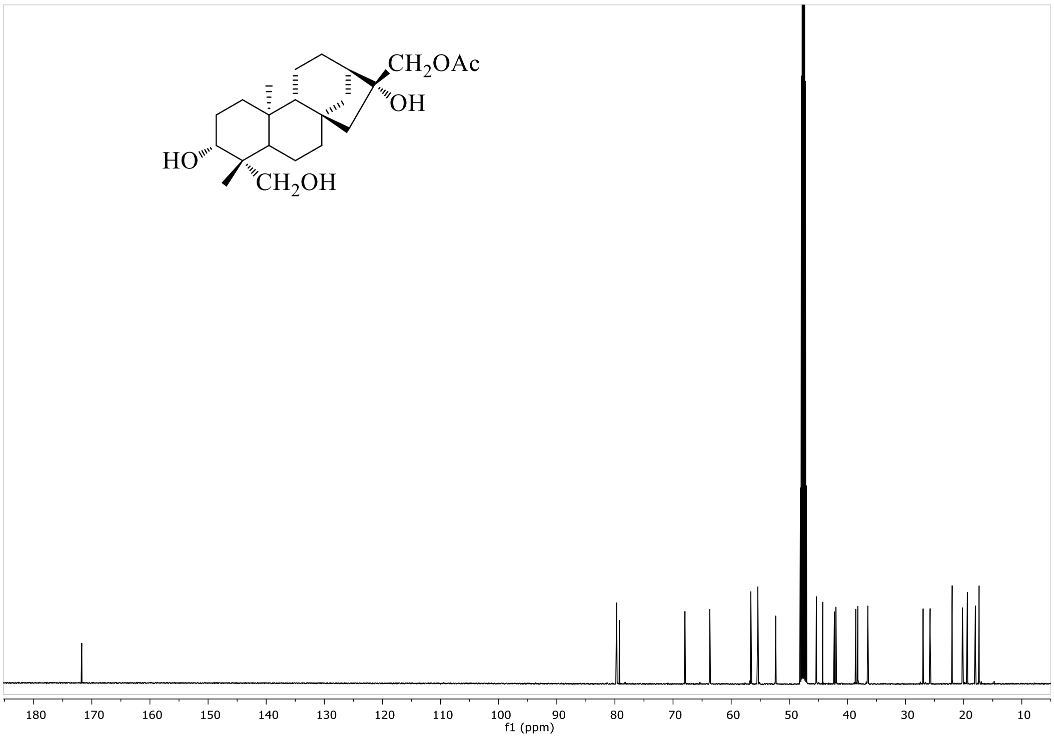

^{13}C NMR spectrum of moquinian B (5) (125 MHz, MeOH)

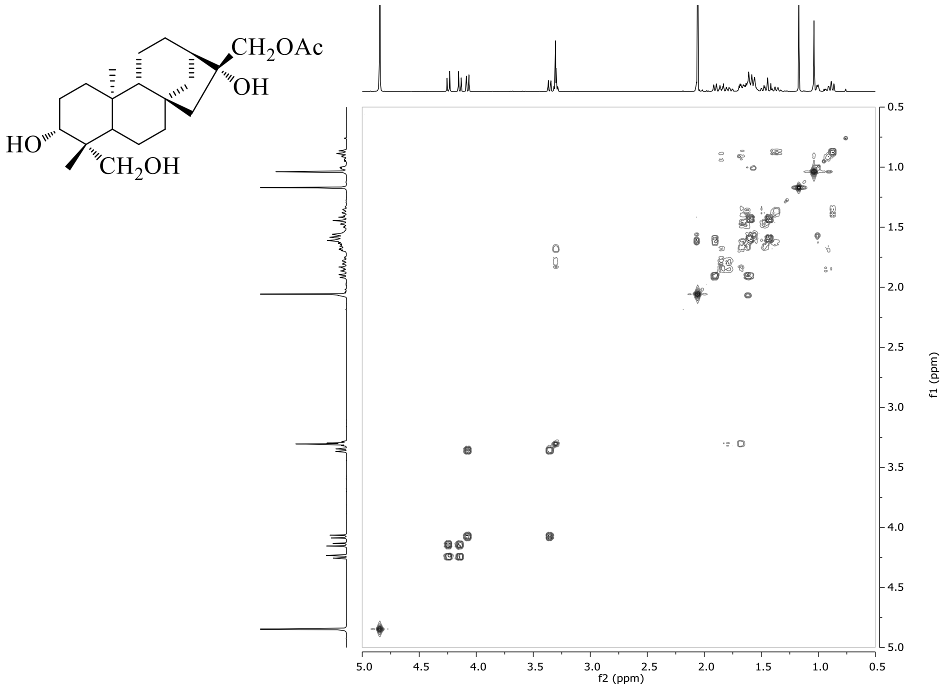

^{1}H-^{1}H COSY spectrum of moquinian B (5) (125 MHz, MeOH)

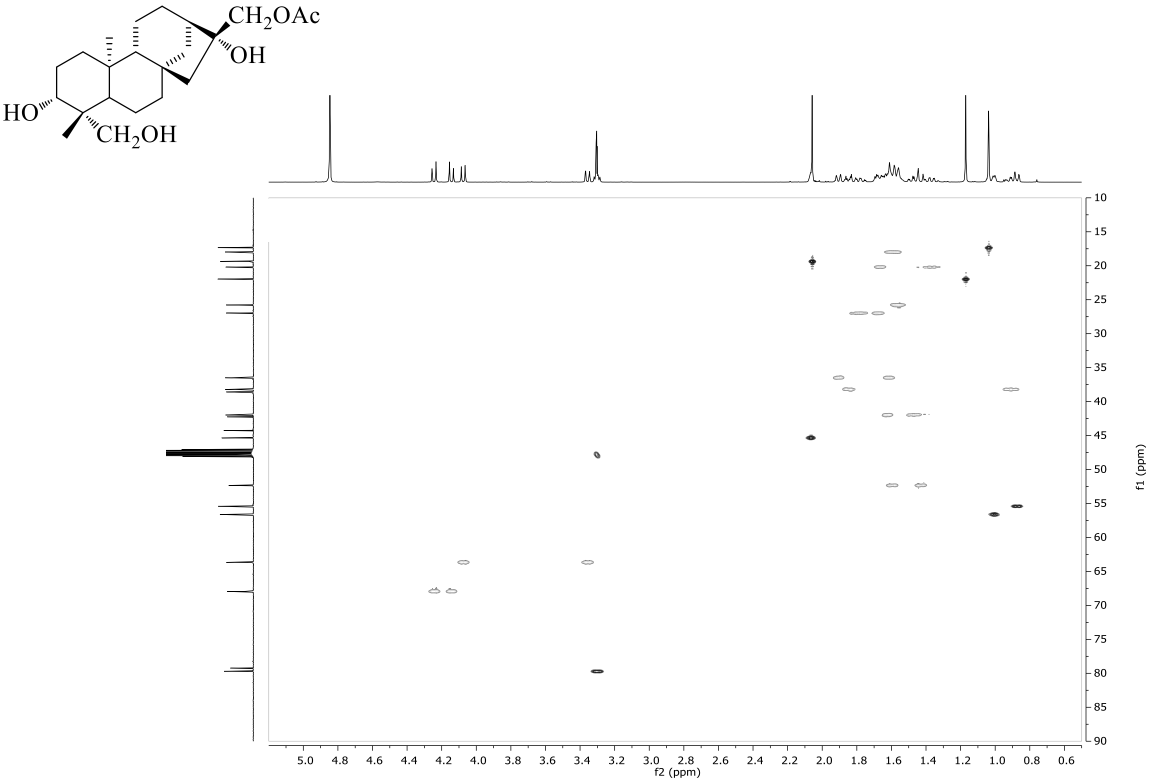

HSQC spectrum of moquinian B (5) (125 MHz, MeOH)

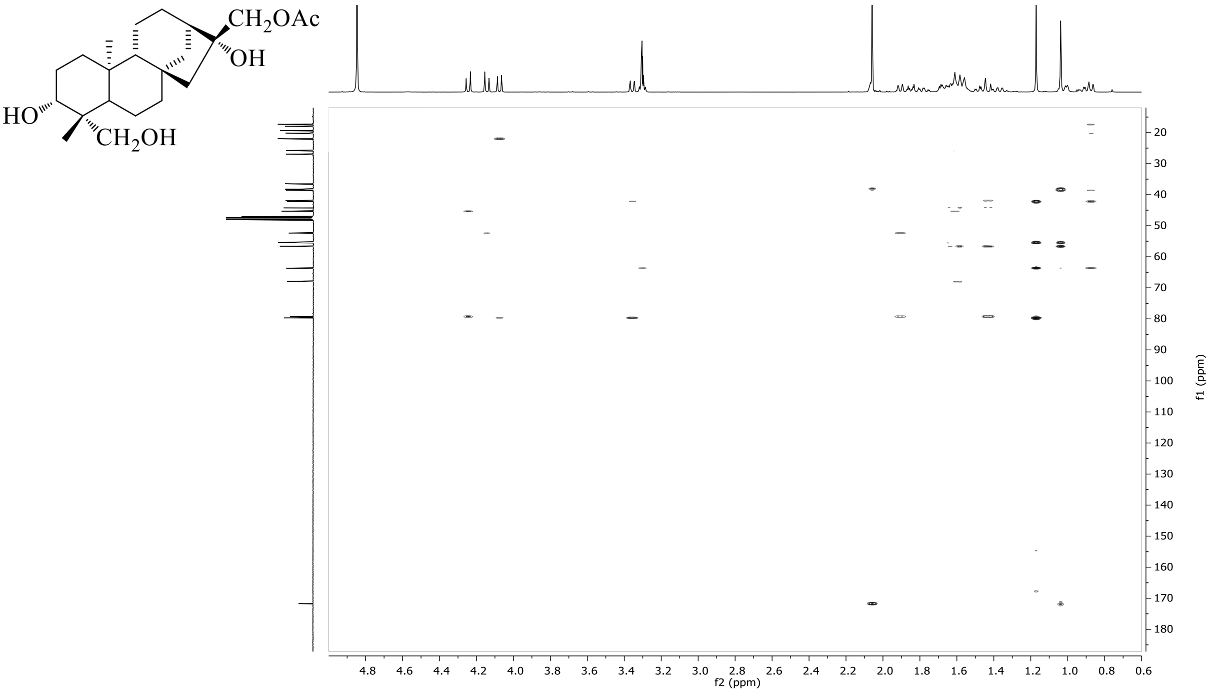

HMBC spectrum of moquinian B (5) (125 MHz, MeOH)

Single Mass Analysis
Tolerance = 1.0 mDa / DBE: min = -1.5, max = 80.0
Element prediction: Off
Number of isotope peaks used for i-FIT = 3

Monoisotopic Mass, Even Electron Ions
159 formula(e) evaluated with 1 results within limits (up to 10 best isotopic matches for each mass)
Elements Used:
C: 0-100 H: 0-100 O: 0-20 Na: 0-1
chon
d5-dll1 433 (4.016)

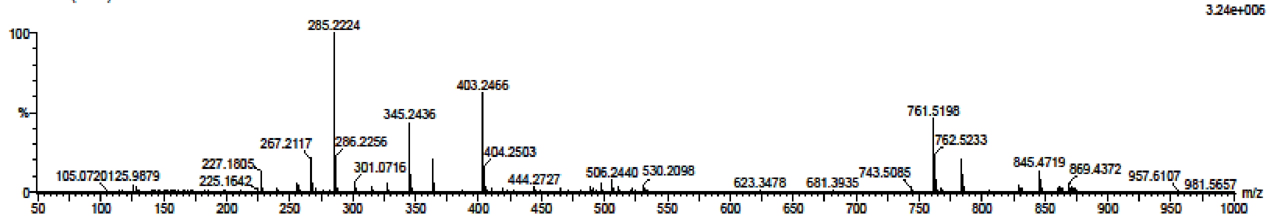

Minimum:				-1.5					
Maximum:		1.0	10.0	80.0					
Mass	Calc. Mass	mDa	PPM	DBE	i-FIT	Norm	Conf(%)	Formula
403.2466	403.2460	0.6	1.5	4.5	1266.1	n/a	n/a	C22 H36 O5 Na

TOF MS spectra of moquinian B (5)
